# Using bone mineral apparent density for BMD adjustment in chronic diseases: Thalassemia an example

**DOI:** 10.22088/cjim.15.3.494

**Published:** 2024-08-01

**Authors:** Mohammad-Reza Mohajeri-Tehrani, Hadis Gerami, Sara Shirazi, Mobina Ostadi, Bagher Larijani, Zohreh Hamidi

**Affiliations:** 1 Endocrinology and Metabolism Research Center, Endocrinology and Metabolism Clinical Sciences Institute, Tehran University of Medical Sciences, 5th Floor, Shariati Hospital, North kargar Avenue, Tehran, Iran; 2 Osteoporosis Research Center, Endocrinology and Metabolism Clinical Sciences Institute, Tehran University of Medical Sciences. No. 10, Jalal-AL-Ahmad St, Chamran HWY, Tehran, Iran

**Keywords:** BMD, Adjustment, Children, Thalassemia, BMAD

## Abstract

**Background:**

Low BMD is a common problem in major thalassaemia patient, but the use of DXA in chronic disease children with smaller bones, has some problems. Utilizing bone mineral apparent density (BMAD) helps in preventing this obstacle. Testing the usefulness of this method in resolving the effects of bone size on BMD by comparing the BMD and BMAD of our thalassemics with results of our healthy ones, is our goal.

**Methods:**

Sample size was 110 cases with mean age of 9.6 ± 4.3 y/o and contained 73 males. Gauge of BMDs done by dual x-ray absorptiometry. Then BMAD was calculated. We did comparison of BMDs and BMADs results of thalassemic children with results of healthy Iranian pediatrics.

**Results:**

Mean of femoral BMD and BMAD, spinal BMD and BMAD was 0.579±0.134 g/cm2, 0.162±0.096 g/cm3, 0.563±0.118 g/cm2 and 0.107±0.015, respectively. When results of 9-18 patients compared with BMDs and BMADs of normal children, BMD of femur and BMD and BMAD of spine of patients found significantly lower (P-values, 0.003, <0.001, <0.001, respectively). BMAD of femur of patients was not significantly different from normals.

**Conclusion:**

When bone mineral density of femur modifies by bone mineral apparent density formula, the remarkable difference between BMD of patients and normals; vanishes. Utilizing bone mineral apparent density helps in interpretation of femoral dual X-ray absorptiometry at least in thalassemic patients. As the results of vertebrae, after modification by calculating BMAD, remains significantly different, we cannot bring forward BMAD for mentioned aim in the spine of thalassemics.

BMD in childhood affects long term bone health and risk of fracture in later life. Some parameters affect the skeleton mineralization such as genetic, gender, nutrition, hormones and mechanical parameters. The gold standard for assessing bone strength and density in adults and children is DXA (1). The basic problematic matter in interpreting the DXA results in pediatrics is the effect of size on bone mineral density, because dual X-ray absorptiometry is a two dimensional method and measures BMD per area unit not per volume (2-4). On the other hand, larger bones have a higher areal bone mineral density. and smaller bone (a common problem in chronic diseases) show lesser BMD (5)**.** So finding ways to calculate real bone density (BMC/bone volume) is under investigation. Calculating bone mineral apparent density (BMAD) is mostly the utilized way to reach this goal (6, 7). 

The BMAD method is proposed based on the supposed geometric relationships between DXA assumed bone area and thickness of bone or one’s height. It is introduced to adjust the size-related effects on BMD results by DXA and estimate of bone density (g/cm3). On the other hand, by dividing BMC by area^1.5 ^in spine and dividing it by area^2^ in femur, near real BMD is calculated. 

Because of the importance of BMAD, reference data in different countries were obtained. In England, USA, Mexico and Korea reference data obtained in children in age range were 4-20 y/o, 5-19 y/o, 5-18 y/o and 10-25 years old, respectively (8-11). Jeddi et al. in Iran obtained such a normal reference in 9-18 y/o ones (12). 

The importance of real bone density estimation is greater in chronic disease. In general, these children have smaller bones due to their developmental problems, and in many cases, due to the underlying disease and its features. Studies of BMAD were performed in some chronic diseases such as X-linked hypophosphatemia, Rett syndrome, osteogenesis imperfecta, turner syndrome, anorexia nervosa, Down syndrome, diabetes, and juvenile arthritis (13-20). 

We planned this project to study thallasemic patients. Thalassemia is the most common monogenic disease in the world and an endemic disease of Iran (in this text, when we mention thalassemia, we mean beta thalassemia major). These patients are at risk of osteoporosis for various reasons such as chronic anemia, transfusions dependent iron overload and secondary hemochromatosis, using chelator agents and endocrinopathies (21-24). 

Osteoporosis was reported in 30 to 50% of them (25, 26). An increase in the risk of osteoporosis increases the risk of pathological fractures and apart from significantly reducing their quality of life, the disease burden increases significantly. Our goal is finding BMAD of Iranian pediatric thalassemic patients and compare the results with the results of normal Iranian children. Previous studies conducted on BMAD in thalassemia major patients and found that age and puberty are important factors in BMAD findings (27, 28).Our study helps testing the effectiveness of BMAD in solving regional BMD problems in another chronic disease, and testing BMAD in thalassemia, an important chronic disease in Iran. Comparing with normal Iranian children shows how far this method can eliminate the effect of small bones in them.

## Methods

**Study participants and research environment:** The participants in this study were 110 beta-thalassemia major 3–18 years (9.6 ± 4.3 y/o) who referred to our research center from 2011-2019. We entered all referred patients, consecutively. A medical history was obtained about any drug or disease that affected BMD. No one was a current or chronic user of systemic steroids. Thalassemics with other major risk factor for osteoporosis were omitted. Short stature was considered when height percentile was less than 3 percentile of normal height for age. Medical ethics code is IR.TUMS.EMRIREC.1395.0075. 

Declaration of Helsinki and its later amendments, considered as guideline. BMD measured by one dual x-ray absorptiometry machine (Hologic, Discovery) and by one operator using pediatric software.

**Statistical analysis:** Lumbar spine BMAD= BMC of L2 _ L4 /area^1.5^ Femoral neck BMAD= BMC of femoral neck/ area^2. ^Due to age of Iranian control group (9-18 y/o), BMDs and BMADs of 9-18 y/o patients were compared with the results of healthy Iranians children of Jeddi et al. ‘s study (12)**.** We used the one sample t-test for comparison of results of two groups. All comparisons were made two-tailed, and statistical significance was set at 5%. SPSS 19 used for statistical analysis. 

## Results

From 110 participants, 36 were females. Short stature was found in 13%. Mean of femoral bone mineral density and bone apparent mineral density and those related to vertebrae was 0.579± 0.134 g/cm2, 0.162±0.096 g/cm3, 0.563 ±0.118 g/cm2 and 0.107±0.015 g/cm3, respectively. BMDs and BMADs of dwarf patients were not different from participants with normal height. Males had significantly higher femur BMD (P= 0.008) and females had significant higher spine BMAD (P= 0.007). When BMD of 9-18 patients were compared with BMDs and BMADs of normal Iranian children, BMD of femur and BMD and BMAD of spine of patients were found significantly lower than normal controls (P-values, 0.003, <0.001, <0.001, respectively). BMAD of femur was not significantly different from normal. You see more detailed characteristics table 1. 

**Table 1:** Characteristics of male and female patients

**Patients** **Characteristics**	**Male**	**Female**	**P-value**
**Height**	131.4	127.4	0.352
**Weight**	30.8	28.0	0.265
Femur BMD*	0.603	0.532	0.008
Femur BMAD**	0.170	0.146	0.220
**Spine BMD**	0.558	0.573	0.537
**Spine BMAD**	0.104	0.112	0.006

*BMD: Bone mineral density; **BMAD: Bone Mineral Apparent Density

## Discussion

As explained in introduction part, the measurement of real BMD (g/cm3) depends on two factors, bone content and volumetric size of bone. This situation is of great importance in pediatrics. If we find low BMD in small for age child, this may be simply due to their small bones, not osteoporosis or mineralization disorders. Wide variation in age at onset and progression of puberty is another problem. So the need to find an adjustment method, seems clear**.** Finding an adjustment method that consider all factors like height, body size, bone volume, puberty stage and bone age is not possible now. In the absence of such gold standard calculating bone mineral apparent density (BMAD) is the most utilized way to reach this goal.

In this study, we examined BMAD in pediatric major thalassemic patients. Short stature was not a determining factor in BMD and BMAD of spine and femur. This is in agreement with findings of study in normal persons (29) though of course not in all of studies (30) . About the effect of gender on BMD, there is controversy. Some authors suggest that there is no significant effect (31)**, **some believe that males are less affected** (**32**), **and another mentioned that females are less affected (33)**. **We found higher femur BMD in males and higher spine BMAD in females. We have no explanation for these findings.

In previous studies, no comparison was done between these children and normal ones. Our study showed that BMD of femur and BMD and BMAD of vertebrae is remarkably lesser than healthy participants but BMAD of femur is not significantly different. It means that when BMD of femur adjusts by BMAD, the significant difference between BMD of normal ones and thalassemic patients,disappears. So we conclude that the use of BMAD for averting the problems of interpretation of femoral DXA in chronic patients -at least in thalassemics- is useful.

We found that after adjustment, spine BMD remains significantly different between normal and thalassemic patients so we cannot recommend BMAD of spine as a standard for BMD adjustment in thalassemic patients. Though our study has limitations, such as no assessment of osteoporosis risk factors, puberty status and endocrine results on BMAD of patients, but sample size (110 thalassemic patients), extensive pediatric age range (from 3-18 y/o) and comparison of BMD and BMAD results with normal cases make findings significant. Wider projects are required to affirm our results.
